# Advantage of Nanotechnology-Based Genome Editing System and Its Application in Crop Improvement

**DOI:** 10.3389/fpls.2021.663849

**Published:** 2021-05-28

**Authors:** Sunny Ahmar, Tahir Mahmood, Sajid Fiaz, Freddy Mora-Poblete, Muhammad Sohaib Shafique, Muhammad Sohaib Chattha, Ki-Hung Jung

**Affiliations:** ^1^Institute of Biological Sciences, Universidad de Talca, Talca, Chile; ^2^Chinese Academy of Agricultural Sciences, Beijing, China; ^3^Department of Plant Breeding and Genetics, The University of Haripur, Haripur, Pakistan; ^4^College of Plant Science and Technology, Huazhong Agriculture University, Wuhan, China; ^5^Graduate School of Biotechnology & Crop Biotech Institute, Kyung Hee University, Yongin, South Korea

**Keywords:** CRISPR, genome editing, nanotechnology, nanoparticles, speedy crop improvement, crop enhancement

## Abstract

Agriculture is an important source of human food. However, current agricultural practices need modernizing and strengthening to fulfill the increasing food requirements of the growing worldwide population. Genome editing (GE) technology has been used to produce plants with improved yields and nutritional value as well as with higher resilience to herbicides, insects, and diseases. Several GE tools have been developed recently, including clustered regularly interspaced short palindromic repeats (CRISPR) with nucleases, a customizable and successful method. The main steps of the GE process involve introducing transgenes or CRISPR into plants via specific gene delivery systems. However, GE tools have certain limitations, including time-consuming and complicated protocols, potential tissue damage, DNA incorporation in the host genome, and low transformation efficiency. To overcome these issues, nanotechnology has emerged as a groundbreaking and modern technique. Nanoparticle-mediated gene delivery is superior to conventional biomolecular approaches because it enhances the transformation efficiency for both temporal (transient) and permanent (stable) genetic modifications in various plant species. However, with the discoveries of various advanced technologies, certain challenges in developing a short-term breeding strategy in plants remain. Thus, in this review, nanobased delivery systems and plant genetic engineering challenges are discussed in detail. Moreover, we have suggested an effective method to hasten crop improvement programs by combining current technologies, such as speed breeding and CRISPR/Cas, with nanotechnology. The overall aim of this review is to provide a detailed overview of nanotechnology-based CRISPR techniques for plant transformation and suggest applications for possible crop enhancement.

## Introduction

**FIGURE 1:**
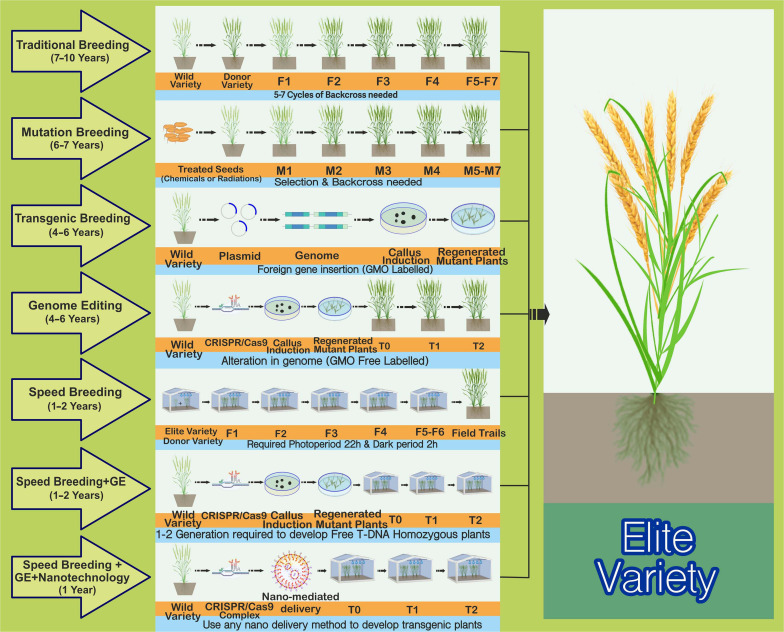
Comparison of the most commonly employed plant breeding mutagenic and time-saving strategies for crop improvement. **Traditional plant breeding** is used to enhance plant characteristics. The complex successive backcrossing and rigorous selection process of the elite recipient’s parent line with a donor line leads to the development of an outstanding progeny with desired traits. This is a time-consuming, laborious, and less-effective technique. **Mutation breeding**, also known as “variation breeding,” refers to seeds being treated with chemicals or radiation to produce mutants with suitable characteristics to develop elite cultivars. It would require 6–7 years to produce desirable outcomes and is also a time-consuming process. Random mutations in the genome are one of the critical drawbacks and disadvantages of this strategy. **Transgenic breeding** has been successfully utilized to improve various crops with different traits by importing a gene of interest from one plant genome to another. These are regarded as genetically modified organisms (GMOs) owing to the insertion of foreign DNA/elements into the genome, and one of the biggest problems with GMOs is their comparative lack of acceptance among the public and a large group of plant scientists worldwide. **Genome editing** (GE) methods, such as the CRISPR/Cas9 method for trait improvement, provide a cost-effective, stable, time-saving, and less laborious solution than other existing techniques. Moreover, these methods can also be used to evade the GMO law, labeling the products as “non-GMO” because of the absence of any foreign DNA. **Speed breeding** that extends the photoperiod (22 h with 2 h of darkness in a 24-h diurnal cycle) improves the flowering time compared with that under normal conditions, potentially achieving four to six generations per year rather than the single generation achieved under normal conditions. Regarding photoperiod, continuous light is another option, but the dark period slightly improves the plant health. The optimal temperature regime (maximum and minimum temperatures) should be applied for each crop. This presents the best strategy for developing elite organic varieties within 1–2 years. The GE technology could also be improved by using speed breeding to establish a transgene-free plant within 1–2 years rather than waiting for an entire season under average growth. Another strategy involving nanotechnology and a combination of speed breeding and GE is proving reliable for speedy crop improvement. Here, plants can be grown under speed breeding conditions, and NPs coated with DNA, RNA, or RNP can deliver CRISPR reagents into meristematic cells. Transgene-free edited plants are obtainable from the edited tissues, either sexually or asexually.

Food safety has become a worldwide issue because of increasing food demand and reducing crop yields resulting from climate change, soil degradation, and crop disease proliferation (Shaheen and Abed, 2018). By 2050, the global population will reach an estimated 9.6 billion, with the demand for staple crops increasing by 60% (Bajželj et al., 2014). Current efforts are focused on sustainably increasing crop yields without the excessive use of pesticides and fertilizers. However, traditional strategies used for crop improvement are laborious, time-consuming, and challenging (Figure 1). Therefore, novel plant breeding technologies equipped with capabilities (gene knock out/in, epigenetic modifications, generation of heritable targeted mutations in specific genomic region) need to be urgently utilized to tackle the drawbacks of the classical plant breeding methods (Chen et al., 2019; Fiaz et al., 2021). In the last decade, there have been major developments in the field of biotechnology, e.g., the advent of third-generation genome editing (GE) techniques, genome sequencing, advancements in plant-based synthetic biology, and bioengineering (Altpeter et al., 2016; Wang et al., 2019; Zhang et al., 2019a). These techniques have been successfully employed to develop elite germplasm, ensuring grain yield, quality, and resistance against biotic and abiotic stresses as well as climate change (Cunningham et al., 2018; Fiaz et al., 2020).

GE techniques have revolutionized biological sciences via precise modifications in the genome of both plants and animals. GE is broadly categorized into three generations: meganuclease (MegaN) and zinc finger nucleases (ZFNs) are first–generation tools, transcription activator-like effector nucleases (TALENs) are second-generation tools, and the clustered regularly interspaced short palindromic repeat (CRISPR)/CRISPR-associated protein 9 Cas9) nuclease system is considered the third-generation tool. Third-generation GEs, e.g., CRISPR/Cas9, CRISPR–CRISPR from *Prevotella* and *Francisella* 1 (Cpf1), base editing, and prime editing, were shown to be powerful tools for the successful modification of genome sequence in a precise and straightforward manner (Puchta et al., 1993; Wright et al., 2005; Christian et al., 2010; Butler et al., 2016; Tang et al., 2017; Yin et al., 2017; Anzalone et al., 2019; Manghwar et al., 2019; Lin et al., 2020). A sequence-specific nuclease catalyzes double-strand breaks at a target region in the genome under consideration. MegaN, a naturally occurring endonuclease discovered in the late 1980s, requires enzymes specific to the targeted sequence and is costly and time-consuming (Townsend et al., 2009). ZFNs were demonstrated in 1996 for the first time as site-specific nucleases for cutting DNA at strictly defined sites. The design of ZFNs is complicated because of the complex interaction of ZFNs among themselves with the risk of off-target mutations (Sander et al., 2011). Furthermore, to increase efficiency, researchers have no other option but to utilize commercially produced ZFNs that are not budget-friendly (Ramirez et al., 2008). TALEN effectors for DNA targeting were realized in 2009. The construction of TALENs is relatively easier and popular than that of ZFNs; however, repetitive sequences in the composition of TALENs can increase the rate of homologous recombination. Both ZFNs and TALENs are the same at the structural and functional level because they harbor the restriction endonuclease *Fokl* (Boch et al., 2009). The classical first- and second-generation GE techniques have drawbacks, and researchers developed a third-generation GE system (Nekrasov et al., 2013). The CRISPR/Cas system is a powerful gene-editing tool that can be used with various model and non-model plant species. It has been used for improving major crops, including rice (*Oryza sativa*; Dong et al., 2020; Fayos et al., 2020), sorghum (*Sorghum bicolor*; Char et al., 2020), tobacco (*Nicotiana tabacum*; Tian et al., 2020), wheat (*Triticum aestivum*; Ferrie et al., 2020; Li et al., 2020), maize (*Zea mays*; Zhang et al., 2020), barley (*Hordeum vulgare*; Zeng et al., 2020), cotton (*Gossypium hirsutum*; Lei et al., 2020), tomatoes (*Solanum lycopersicum*; Santillán Martínez et al., 2020), soybeans (*Glycine max*; Wang L. et al., 2020), and rapeseed (*Brassica napus*; Zheng et al., 2020). However, the safe, efficient, and precise time-saving delivery of CRISPR components remains a challenge (Rui et al., 2020). Speed editing strategies have been proposed to address this challenge recently. A web tool has been developed by Hong et al. (2020) to accelerate speed editing strategies to achieve 2% genetic gain in crop productivity (2050 food demand challenge). The powerful GE technology CRISPR/Cas has facilitated functional genomic studies of several crops with simplicity and accuracy. However, genomic research has become congested because of functional redundancies in the genome, ultimately masking the phenotypes of knockout mutants by functional compensations and redundancies. To cope with this concern, an intuitive tool called CRISPR was applied to a functional redundancy inspector to accelerate functional genomics in rice (CRISPR Applicable Functional Redundancy Inspector [CAFRI]-Rice; cafri-rice.khu.ac.kr). The tool is based on a phylogenetic heatmap that can estimate the similarity between protein sequences and expression patterns. This CAFRI-Rice-based target selection for CRISPR/Cas9-mediated mutagenesis has accelerated functional genomic studies in rice; moreover, it can also be easily expanded to other plant species (Ahmar et al., 2020b; Hong et al., 2020).

Conventional biomolecule delivery methods in plants have critical drawbacks, such as low efficiency of gene transmission, narrow species range for applocation, limited cargo types, and tissue damage. Nanotechnology advancements have created opportunities to overcome limitations in conventional methods: nanoparticles (NPs) are promising for the species-independent passive delivery of DNA, RNA, and proteins (Cunningham et al., 2018). There are hundreds of transformation methods. Of them, the two primary genetic transformation methods used in plants are typically genotype-specific for gene delivery (Stewart et al., 2011). The first method, *Agrobacterium*-mediated transformation (AMT), is widely used for incorporating of target DNA to the nuclear genome and is available for a limited number of plant species. The AMT method leads to random DNA integration, disrupting endogenous plant genes and variation in gene expression arising from the inserted sites (Niazian et al., 2017). The second method, the biolistic delivery of DNA, involves a high-pressure gene gun that directly targets plant tissues, randomly integrating DNA into the chromosomal region across cell walls and membranes. This leads to the destruction of tissues and multiple insertions in random portions of the plant genome (Toda et al., 2019). Thus, plant transformation presents a major bottleneck for GE capacity. Therefore, the delivery method of the biomodifier-conjugated complex to plant cells remains a topic of study for many scientists to develop new strategies for transformation with ease, robustness, and significant efficiency.

Nanotechnology is modern science, and molecular biology has significantly benefited from research in this subject. The inclusion of nanotechnology in the development of genetically modified (GM) organisms (GMOs) represents a powerful tool involving the use of NPs as nanocarriers by producing a binding complex with biomodifier molecules (CRISPR/Cas system) and delivery into plant cells (Abd-Elsalam, 2020; Demirer et al., 2021). The implementation of nanotechnology in the existing molecular technologies could also create a forum for overcoming barriers to produce genetically engineered plants as well as for biotransformation (Cunningham et al., 2018; Gad et al., 2020). Nanomaterial (NM) engineering has emerged as a cutting-edge technology to develop crops for sustainable farming systems (Panpatte et al., 2016). The development of nanodevices and NMs can reduce the effect of significant stresses on food and energy production while maximizing the use of limited resources, including water or nutrients (Giraldo et al., 2019).

Nanobiotechnology techniques have improved the precision of plant breeding in generating exciting new possibilities for gene selection and transition, reducing the time required to remove unwanted genes and enabling the breeder to access essential genes from large plantations (Pérez-de-Luque, 2017). The magnetofection of the bioconjugated complex of transgene and NPs have successfully been reported in dicots (Zhang et al., 2019b). Here, nanotechnologies that enable the force-independent supply of DNA without integrating transgenes indicate that the transient expression of CRISPR techniques can be used in most countries for permanent GM-free GE (Figure 1; Li et al., 2018). The main steps in GE include introducing transgenes or CRISPR/Cas9 into plants via specific gene delivery systems (Hansen and Wright, 1999; Grunewald et al., 2013). Gene transformation delivery using nanotechnology is superior to traditional biomolecular approaches mainly because the former enhances the transformation efficiency for both temporal (transient) and permanent (stable) genetic modifications in various plant species (Serag et al., 2013; Vanhaeren et al., 2016; Novák et al., 2017). Hence, nanotechnology can reduce uncertainty and help coordinate the management strategies of agriculture using molecular production approaches as an alternative to conventional technologies (Figure 1).

NPs have begun to facilitate and enhance GE via an efficient and targeted delivery of plasmids, RNA, and ribonucleoproteins (RNPs). In mammalian cells, NPs are routinely used for the efficient, direct cytosolic/nuclear delivery of Cas–RNPs in many cell types (Mout et al., 2017). RNP delivery has been shown to greatly reduce off-target effects compared with plasmid-based CRISPR systems (Liu et al., 2017). However, in plants, the cell wall has hindered the development of an analogous system that can passively deliver GE cargo into mature plants. Thus, there remains much potential for designing NP carriers (DNA, RNA, and proteins) with diverse cargo-loading capabilities and optimal geometry/chemistry to efficiently bypass the cell wall and membranes in dense plant tissues without external aid. A previous work (Burlaka et al., 2015) showed that some NP formulations undergo passive internalization in plants with DNA, RNA, or protein cargo (Demirer et al., 2018).

With the discoveries of several advanced technologies, the need to develop short-term crops to feed the ever-increasing population daily remains pertinent. This review presents a concept to hasten the existing crop improvement technologies by combining them to produce efficiently and high-yield improved crop plants. Speedy crop improvement can be achieved using CRISPR under speed editing strategies via NMs combined with speed breeding (For speed breeding, see the detailed review by Watson et al. (2018). This review first describes the nanobased delivery methods and plant genetic engineering techniques along with NP-mediated genetic engineering challenges in addition to the speedy crop improvement concept. Further challenges and the future use of nanotechnology are also described in detail. The overall goal is to provide a comprehensive summary of plant-related CRISPR techniques that incorporate nanotechnology and consider future application prospects for crop improvement.

## Conventional Plant Biomolecule Delivery Approaches and Their Limitations

The genetic transformation of plants involves two main steps: genetic cargo delivery and the regeneration of transformed plants. Here, the regeneration capacity depends on the biomolecule delivery method employed and whether a stable transformation is desired (i.e., constitutive or transient; Cunningham et al., 2018). Various biological, chemical, and physical methods are available to deliver genetic materials into plant cells, including the aforementioned AMT, viral-mediated transformation, polymer-mediated delivery (e.g., polyethylene glycol [PEG)]), particle bombardment (gene gun-mediated or biolistic transformation), and electroporation (Ahmed et al., 2018; Mohammed et al., 2019; Shin et al., 2019; Tian et al., 2019; Imai et al., 2020; Ozyigit, 2020). The features of the conventional transformation methods are summarized in Table 1. Gene gun-mediated transformation and AMT are among the most efficient and commonly utilized gene delivery methods for plant-related genetic transformation. These methods have been adopted for various crops, including soybeans (Li et al., 2017; Zhao et al., 2019; de Melo et al., 2020), sorghum (Che et al., 2018; Liu G. et al., 2019; de Melo et al., 2020; Sharma et al., 2020), maize (Char et al., 2017; Anand et al., 2018; Raji et al., 2018), sugarcane (Mayavan et al., 2015; Wu et al., 2015; Dessoky et al., 2021), wheat (Liang et al., 2018; Kumar et al., 2019; Zhang et al., 2019c), and rice (Endo et al., 2015; Ling et al., 2016; Feng et al., 2017).

**TABLE 1:** Physical, biological, and chemical conventional transformation methods in plants.

Delivery method	Plant–host range	Target tissue	Advantages	Adverse effects or disadvantages	References
**Physical**					
Electroporation	Unrestricted	Pollen grains, protoplasts, and meristems	Simple, fast, and inexpensive as well as wide a plant–host range	Non-specific transport of material and damage to the target tissue	Cunningham et al., 2018; Keshavareddy et al., 2018; Sangeetha et al., 2019; Ramkumar et al., 2020
Biolistic	Unrestricted	Microspores and intact tissue	Suitable for large-sized genetic cargo	Scrambled and multiple integrations, damage to the target tissue, and specialized equipment is required	Altpeter et al., 2005; Gao C. et al., 2008; Cunningham et al., 2018; Lacroix and Citovsky, 2020; Ramkumar et al., 2020
**Biological**					
*Agrobacterium*	Restricted	Immature tissues (e.g., callus and meristems) and cells	Stable gene integration, high-efficiency transformation, and no specialized equipment is required	High host specificity and limited to DNA cargo	Ishizaki et al., 2008; Sood et al., 2011; Krenek et al., 2015; Cunningham et al., 2018; Keshavareddy et al., 2018
Viral vectors	Restricted	Immature tissues (e.g., callus and meristems) and cells	Easy to set up, quick, and affordable	High host specificity and limited cargo size	Jones et al., 2009; Cunningham et al., 2018; Keshavareddy et al., 2018
**Chemical**					
Polymers (polyethylene glycol)	Unrestricted	Protoplasts	Various genetic cargo types (DNA, siRNA, and miRNA) and economical procedure	High concentrations induce toxicity	Cunningham et al., 2018; Yu et al., 2020

Despite more than three decades of development, plant transformation and regeneration remain a challenge in several crop plants. AMT has proven to be more efficient for dicots than monocots (Sood et al., 2011; Van Eck, 2018; Mohammed et al., 2019) and is limited to a specific plant–host range (Cunningham et al., 2018; Demirer et al., 2019a). For example, AMT efficiency tends to be highly variable (6–99%) depending on the variety and subspecies of rice (Mohammed et al., 2019). In general, monocots are considered recalcitrant for *Agrobacterium tumefaciens*-mediated transformation (Sood et al., 2011; Hiei et al., 2014; Hofmann, 2016; Mookkan et al., 2017). However, the AMT method has undergone several changes to optimize monocot genetic modification (Hiei et al., 2014; Singh and Prasad, 2016; Anand et al., 2018). For example, the use of hypervirulent strains with standard or superbinary vectors has been shown to improve the transformation efficiency of the AMT method (Shrawat and Lörz, 2006; Singh and Prasad, 2016). In addition, Anand et al. (2018) developed a ternary vector system that has a high transformation frequency in an elite maize inbred line.

One of the advantages of the biolistic method over AMT is related to the variety of species transformed by the former (Matsumoto and Gonsalves, 2012; Cunningham et al., 2018). In general, this method is preferred for rapid assays using transient expressions, such as protein localization, the functional analysis of promoters, and transcription factor characterization (Lenka et al., 2015, 2018; Wang et al., 2018). The particle bombardment method enables the delivery of DNA sequences > 150 kb, albeit with the possible compromise in DNA integrity (Chandrasekaran et al., 2020). Moreover, gene gun-mediated plant transformation can result in scrambled and multiple integrations (Altpeter et al., 2005; Gao C. et al., 2008). Viruses have been used as a vector to introduce foreign genes into various crops (Meziadi et al., 2017; Bouton et al., 2018). In general, viral vector systems are developed for transient expression analyses.

Of note, several viral vectors have been specifically developed to transform plants recalcitrant to AMT, such as monocots (Bouton et al., 2018). Meanwhile, virus-mediated transformation is limited by the virus’ host specificity (Jones et al., 2009). Electroporation is less frequently used than other plant transformation methods, whereas an efficient transformation has been achieved in terms of monocots and dicots (Barampuram and Zhang, 2011; Ozyigit, 2020). Similar to viral vector-mediated transformation, electroporation-mediated transformation has largely been used for transient analyses and the investigation of gene functions at the cellular level (Ramkumar et al., 2020).

Along with biolistic methods, PEG-mediated transformation is one of the most commonly used methods for introducing genetic cargo into chloroplasts (Yu et al., 2020). This method enables the carrying of several genetic cargo types, such as DNA and RNAs (small interfering RNA [siRNA] and miRNA; Cunningham et al., 2018). However, it requires regeneration from protoplasts, which is highly challenging because of the limited number of plant species amenable to protoplast regeneration.

Traditional biomolecule delivery methods have several drawbacks, including limited cargo type, narrow species range, low efficiency, and the potential for tissue damage. Novel strategies are therefore needed for efficient gene delivery in crop plants. Tissue culture and regeneration steps are the principal constraints in plant transformation. Clough and Bent (1998) developed the floral dip method that involves directly dripping flower buds into an *Agrobacterium* suspension (or an *Agrobacterium* inoculum is dropped onto the buds) while avoiding cell or calli culture. This plant transformation method has been adopted for several important crops, including maize (Mu et al., 2012), rice (Rod-In et al., 2014; Ratanasut et al., 2017), and rapeseed (Li et al., 2010). However, as noted by Imai et al. (2020), the existing protocols can involve low reproducibility.

## Advanced Plant Biomolecule Delivery Approaches Via the Application of Nanobiotechnology

Nanotechnology-based methods have been proposed as inexpensive, easy, and robust techniques to transfer genes or other molecules into plants with high efficiency and low toxicity (Chandrasekaran et al., 2020). Nanotechnology has significantly impacted various research fields, including medicine, energy, and manufacturing. Nanotechnology-based methods have been used to deliver biomolecules and chemicals into cells in both plant and mammalian cell systems (Chang et al., 2013; Wang et al., 2014; Mahakham et al., 2017; Fortuni et al., 2019); however, compared with the mammalian cell delivery process, NP-mediated plant biomolecule delivery has proven to be more challenging because of the presence of the natural barrier provided by the cell wall (Mao et al., 2019). It has been suggested that the use of NPs enables an efficient plant transformation because NPs protect the genetic cargo from cellular enzymatic degradation (e.g., nucleases; Finiuk et al., 2017; Joldersma and Liu, 2018). NPs for gene delivery are classified according to the base material used and include carbon-based NPs, silicon-based NPs, metallic NPs, and polymer-based NPs. Each NP type delivers different genetic cargos. For example, carbon nanotubes (CNTs) can carry RNA and DNA (Bates and Kostarelos, 2013; Karimi et al., 2015), but metallic NPs can only deliver DNA as genetic cargo (Zhao et al., 2017). In addition, silicon-based NPs can carry DNA and proteins, whereas polymeric NPs (e.g., PEG and polyethyleneimine) can transfer encapsulated RNA, DNA, and proteins into cells (Silva et al., 2010; Moon et al., 2011; Su et al., 2011; Hasanzadeh Kafshgari et al., 2015; Zhou et al., 2018).

**FIGURE 2:**
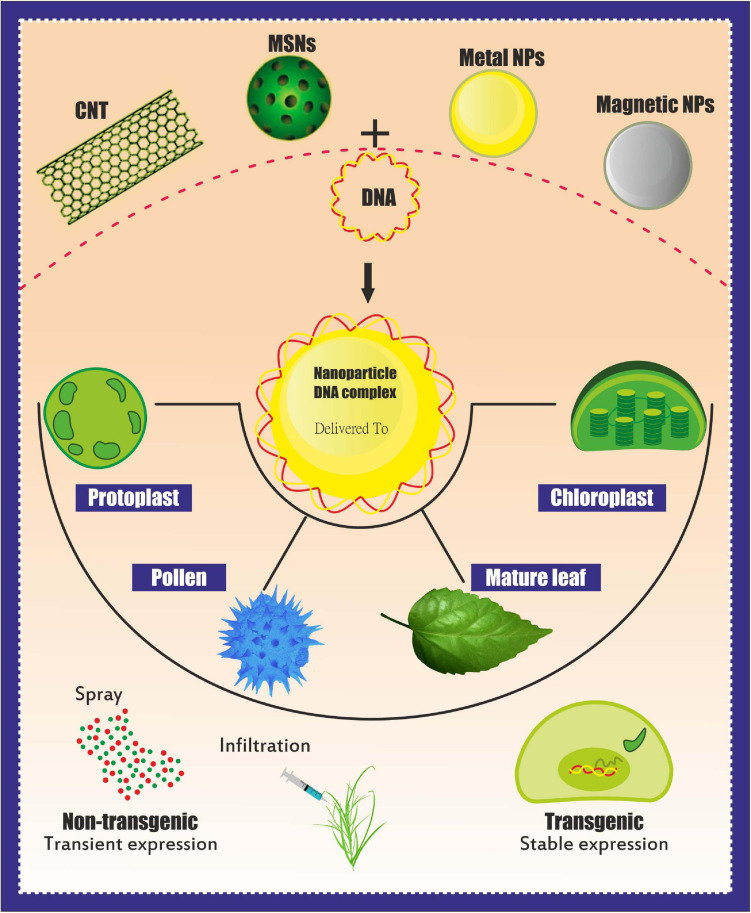
Different-shaped nanoparticles (NPs) for use as genetic cargo for genome editing. The shape and size can be engineered to bind specific biomolecules to produce the most stable bioconjugate complex. NPs can also be used in force-free delivery, i.e., using magnetic properties and electric field usage for penetration (Jat et al., 2020).

Overall, NPs should be capable of crossing the cell wall and localizing into organelles. Cationic NPs are preferred for plant gene delivery because this NP type can bind to the plant cell wall (negatively charged) and perform gene transfer (Albanese et al., 2012), whereas CNT NPs have been used to deliver plasmid DNA into various crops (Table 2 and Figure 2). However, NPs generally require additional physical methods (e.g., magnetoinfection and electroporation) for gene delivery into plant cells. By contrast, NPs, such as silicon carbide whiskers (SCW) and mesoporous silica NPs (MSN), have been effectively used to transfer genes into the plant without using other physical methods (Chang et al., 2013). Here, SCW-mediated transformation has been successfully used to transform tobacco (Golestanipour et al., 2018).

**TABLE 2:** Nanoparticle (NP)-mediated transformation methods used for various crops.

NP type	Genetic cargo	Crop	References
CNTs	DNA plasmid	Arugula	Demirer et al., 2019a
	DNA plasmid	Wheat	Demirer et al., 2019a
	DNA plasmid	Cotton	Demirer et al., 2019a
	DNA plasmid	Tobacco	Demirer et al., 2019a
	DNA plasmid	Tobacco	Burlaka et al., 2015
	DNA plasmid	Arugula	Kwak et al., 2019
	DNA plasmid	Tobacco	Kwak et al., 2019
	DNA plasmid	Spinach	Kwak et al., 2019
Silicon carbide whiskers–carbon nanotubes	DNA	Tobacco	Golestanipour et al., 2018
Gold NPs	DNA	Rice	Wu et al., 2011
	DNA plasmid	Rapeseed	Hao et al., 2013
Gold NPs–mesoporous silica	DNA plasmid	Tobacco	Torney et al., 2007
	DNA plasmid	Maize	Torney et al., 2007
	DNA	Onion	Martin-Ortigosa et al., 2012
	DNA	Maize	Martin-Ortigosa et al., 2014
Zinc NPs	DNA plasmid	Tobacco	Fu et al., 2012
Polymer NPs	siRNA	Tobacco	Silva et al., 2010
Clay nanosheets	dsDNA	Cowpea	Mitter et al., 2017
	dsDNA	Tobacco	Mitter et al., 2017

Nonetheless, in general, the SCW method has one disadvantage compared with other NP-mediated plant transformation in that an adequate protocol is required for plant regeneration from cell cultures. Polymer NPs can also deliver nucleic acids into plant cells. Of note, Silva et al. (2010) used polymer NPs to introduce siRNA into tobacco protoplasts, providing an alternative gene knockout mechanism in plant cells.

Several NPs can penetrate the cell wall (e.g., CNTs and mesoporous silica), whereas other NPs require chemical or physical pretreatments, such as gold NPs and magnetic NPs (MNPs), for genetic cargo delivery into the cells. Meanwhile, NP-mediated passive delivery has been reported with tobacco (Burlaka et al., 2015; Mitter et al., 2017; Golestanipour et al., 2018; Kwak et al., 2019), cowpea (Mitter et al., 2017), and arugula crops (Kwak et al., 2019). NPs and other new materials might serve as useful vehicles for editing systems (Gao, 2021). Working within this context, Hamada et al. (2017) proposed a method involving plant bombardment in which the shoot apical meristems of wheat were used as the target tissue (Imai et al., 2020). In this study, gold particles coated with the green fluorescent protein gene construct were delivered into the L2 cell layer of the shoot apical meristems of wheat. This approach provided a stable transformation in wheat without embryogenic callus culture and can be applied to other crops that have not been successfully transformed via the conventional methods.

## Role of Nanotechnology in Agriculture

Current farming techniques, established during the green revolution, have proven to be largely untenable within the backdrop of the increasing population and climate change (Lowry et al., 2019). Nanotechnology presents reliable solutions for tenable farming, such as encompassing effective pest management and nutrient use, decreasing the impact of environment in food production, and alleviating the effect of climate change (Hofmann et al., 2020). Plant nanotechnology is a flourishing domain in which engineered NMs have been established for analyzing plant functions (Wang et al., 2016, 2019; Giraldo et al., 2019; Kah et al., 2019; Lowry et al., 2019). Meanwhile, NMs are becoming a convenient medium for introducing biomolecules in plants and can be modulated to direct their translocation and distribution in plant cells and organelles (Torney et al., 2007; Kwak et al., 2017, 2019; Demirer et al., 2019b).

Thus far, various NMs have been assessed, including nanofertilizers (employing a thin coating of NMs on plant nutrients and delivering in the form of nanosized emulsions), nanopesticides (tiny molecules that are the only constituent of pest control derivatives and/or entraps the active constituent of pesticide into a protective nanocarrier), and nanobiosensors (nanobiosensor synthesized from the combination of nanotechnology and biosensors, equipped with immobilized bioreceptor probes; e.g., antibodies and enzyme substrate; Usman et al., 2020). Crop production and soil health can be enhanced using different types of NM. Nanofertilizers are regarded as micronutrients or macronutrients and act as transporters for added substances via the incorporation of minerals for the nutrients (Kah et al., 2018). DeRosa et al. (2010) stated that nanofertilizers are also effective in confining nutrients inside the NMs and Kah et al. (2018) have observed an 18%–29% increase in the efficiency of nanofertilizers compared with that of synthetic fertilizers. Iron, manganese, zinc, copper, molybdenum, and silver can be used to improve transportation systems, thereby enhancing the assimilation and efficiency of synthetic fertilizers (Liu and Lal, 2015). The different doses of silver NPs significantly enhance the rate of seed germination in maize, *Citrullus lanatus* (watermelon), and *Cucurbita pepo* L. (pumpkin) crops by having a small toxic impact leads to seed germination (Acharya et al., 2020; Wang F. et al., 2020). Meanwhile, *Lactuca sativa* (lettuce) germination is often improved via titanium dioxide NM electrospraying, although studies have revealed that NMs can remarkably decrease fertilizers’ application in the face of both soil and foliar application, thereby enhancing the efficacy and reducing discharge into the environment compared with synthetic formulations (Adisa et al., 2019).

Synthetic pesticides can be replaced with nanopesticides with a higher potential capacity. The gradual degradation and precise discharge of active components with appropriate NMs can enhance pest management efficacy over long periods (Chhipa, 2017). Therefore, nanopesticides are vital for the effective and tenable management of various pests and can reduce the usage of agrochemicals and, as such, mitigate the existing environmental hazards. These pesticides behave differently from synthetic pesticides, which enhance their efficiency (Kah et al., 2019). The dissolving power of active components could enhance the movement and degradation of soil-inhabiting microorganisms. Furthermore, NP-based pesticides improve the solubility of aluminum and are less hazardous to the environment than synthetic pesticides (Kah and Hofmann, 2014).

In general, nanopesticides bind water and energy as they are released to a small extent and less frequently than synthetic pesticides. They also improve pesticide efficacy and crop production because of greater yields and lower input costs by decreasing waste and labor costs. NPs also exhibit a well-organized antimicrobial activity against viruses and bacteria. Silver, copper, and aluminum are considered vital inorganic NPs with good pesticide properties (Gogos et al., 2012; Kim et al., 2012; Stadler et al., 2012). The efficacy of herbicides can be increased via nanoherbicides that generally anticipate biodegradable polymeric components. For example, poly(-caprolactone) is widely used to contain atrazine owing to its better physiochemical properties and greater bioaccessibility and biocompatibility (Abigail and Chidambaram, 2017). Synthetic chemicals can be introduced in hosts using a conveyer system based on CNTs (Raliya et al., 2013) after targeting a decrease in the number of chemicals discharged into the environment that may damage other plant cells (Hajirostamlo et al., 2015).

**FIGURE 3:**
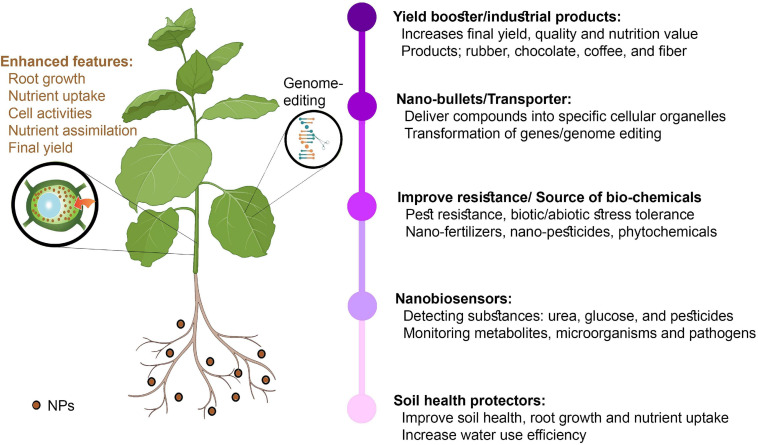
Applications of nanotechnology in plant breeding and agricultural production.

Nanobiosensors are more substantial and are associated with next-generation sensors that detect different elements at ultra-low concentrations via a physiochemical transducer (Scognamiglio, 2013). In short, nanobiosensors can enable plant protection by allowing plants to converse with farmers (Giraldo et al., 2019; Wang et al., 2019), which could help ensure timely decision-making to improve crop productivity via appropriate water, land, fertilizer, and pesticide management. Nanobiosensors have a longer shelf-life than older-generation sensors owing to their greater stability and sensitivity, fast electron kinetics, and higher surface-to-volume ratio (Scognamiglio, 2013). Different nanosensor types have been used in plants, including plasmonic, fluorescence resonance energy transfer-based, carbon-based electrochemical, nanowire, and antibody nanosensors. They can be used to detect substances, such as urea, glucose, and pesticides, monitor metabolites, and detect various microorganisms or pathogens (Rai et al., 2012). Using different NMs, the delay in plant nanotechnology could be controlled. This could be achieved by using smart NMs and NPs, which could ultimately revolutionize the farming industry (Table 3 and Figure 3).

**TABLE 3:** Nanomaterials (NMs) regarded as beneficial for various agricultural crops.

NM	Plant	Application	Impact	References
Ag	Rice, brown mustard, maize, watermelon, summer squash, and radish	Interactions of NPs in plants	Stimulated growth in summer squash and watermelons, stimulated shoot and root length in brown mustard, enhanced photosynthetic efficiency in brown mustard, toxic to maize root growth, and reduced seedling growth in radishes	Sharma et al., 2012; Almutairi and Alharbi, 2015
Au	*Arabidopsis*, flame lily, barley, rice, and tomato	Interactions of NPs in plants and imaging	Not toxic to tomato and barley, enhanced germination and vegetative growth in flame lily, and stronger NP accumulations in roots	Zhu et al., 2012; Gopinath et al., 2014; Dan et al., 2015; Avellan et al., 2017; Milewska-Hendel et al., 2017
CaCO_3_	Peanut	Nutrient solution	Enhanced plant biomass and yield	Xiumei et al., 2005
Ca_5_(PO_4_)OH	Soybean	Nutrient solution	Improved biomass, growth, and yield	Liu and Lal, 2014
Cu	Lettuce, cucumber, mung bean, wheat, and sorghum	Interactions of NPs in plants	Increased total nitrogen, shoot and root length, reduced total biomass, bioaccumulation and toxicity in wheat, mung bean, and sorghum as well as higher NP accumulation and gene deregulation in the roots of cucumber	Lee et al., 2008; Shah and Belozerova, 2009; Mosa et al., 2018
CdSe/ZnS QDs	Onion, *Arabidopsis*, and alfalfa	Interactions of NPs in plants, imaging, fluorescent detection, and nanobiosensors	Biosensors help in pathogen detection, increased reactive oxygen species (ROS) production, and decreased viability of cell and root growth	Santos et al., 2010; Rad et al., 2012; Koo et al., 2015; Modlitbová et al., 2018
CuO	*Arabidopsis*, rice, wheat, and cucumber	Plant genetic engineering	Cu increased the essential nutrients in plant growth, enhanced ROS production, and reduced shoot and root length	Shi et al., 2014; Wang et al., 2016; Mosa et al., 2018
Chitosan	Wheat and tea	Nanofertilizers, nanoherbicides, and plant genetic engineering	Stimulated plant growth, biocompatible and biodecomposing material, antimicrobial activity	Chandra et al., 2015; Aziz et al., 2016; Islam et al., 2017; Malerba and Cerana, 2018
Dendrimer	Bentgrass	Plant genetic engineering	Endosomal escape in DNA delivery	Pasupathy et al., 2008; Kretzmann et al., 2017
Fe_3_O_4_	Soybean, wheat, and maize	Interactions of NPs in plants and nanofertilizers	Enhanced chlorophyll content in soybean, improved plant height and leaf area in wheat, and improved visible brown spots on leaves of maize	Rãcuciu and Creangã, 2009; Ghafariyan et al., 2013; Fathi et al., 2017
Fullerene	Summer squash, soybean, bitter gourd, poplar, tomato, and maize	Delivery of drugs in agriculture	Decreased accumulation of pesticides in maize, soybean, tomato, and summer squash; enhanced biomass and yield in bitter gourd; and increased uptake of trichloroethylene in poplar	Ma and Wang, 2010; De La Torre-Roche et al., 2013; Kole et al., 2013
Liposomes	Benth and tomato	Delivery of nutrients and DNA	Improved delivery of DNA and cell targeting as well as increased protection of nucleic acids	Karny et al., 2018
Mg	Black-eyed pea	Nanofertilizers	Improved chlorophyll content as well as improved plasma membrane stability and yield	Delfani et al., 2014
Mn	Mung bean and chickpea	Interaction of NPs in plants	Enhanced shoot and root length as well as improved chlorophyll and carotenoid contents	Pradhan et al., 2013
Mo	Chickpea	Interaction of NPs in plants	Improved antioxidant metabolism and enhanced nodule number and biomass	Taran et al., 2014
MSNs	Onion, tobacco, and maize	Plant genetic engineering, delivery of pesticides, and nanofertilizers	Control in chemical and nucleic acid release	Torney et al., 2007; Martin-Ortigosa et al., 2014; Rastogi et al., 2019
MWCNTs, SWCNTs	Cotton, benth, tobacco, rice, tomato, rocket salad, *Arabidopsis*, barley, cucumber, ryegrass, rapeseed, and maize	Plant genetic engineering	Improved growth and metabolic activity in tobacco; increased germination, growth, and flowering of tomato; improved delivery of DNA in rocket salad, cotton, and tobacco; enhanced root growth in cucumber, ryegrass, maize, and rapeseed; and apoptosis and chromatin condensation in rice and *Arabidopsis*	Lin and Xing, 2007; Cañas et al., 2008; Shen et al., 2010; Khodakovskaya et al., 2013; Lahiani et al., 2013, 2016; Serag et al., 2013; Demirer et al., 2019a
*SiC* whiskers	Cotton	Plant genetic engineering	Improved genetic transformation	Asad and Arshad, 2011
TiO_2_	*Arabidopsis*, rice, and spinach	Nanofertilizers	Enhanced nitrogen metabolism and plant growth of spinach and improved seed germination	Gao F. et al., 2008; Kurepa et al., 2010; Liu J. et al., 2019
ZnO	Mung bean, chickpea, onion, *Arabidopsis*, rapeseed, cucumber, lettuce, ryegrass, rice, radish, and maize	Nanopesticide micronutrient delivery	Reduced flowering time and yield in onion and improved plant growth, seed germination increased, inhibition of root growth in rapeseed, ryegrass, radish, lettuce, cucumber, and maize at higher application rates	Lin and Xing, 2007; Mahajan et al., 2011; Zhao et al., 2013; Laware and Raskar, 2014
SiO_2_	*Arabidopsis*	Interaction of NPs in plants	SiO_2_ NPs have the potential to serve as an inexpensive, highly efficient, safe, and sustainable alternative for plant disease protection	El-shetehy et al., 2021

*The abbreviations of NMs have been described in the main text.

The focused NP distribution mechanisms of cellular organelles have been highly successful. However, other plant sections do not influence NP particles because they function as silent bullets to release compounds into specific cellular organelles (de Oliveira et al., 2014; Saranya et al., 2019). Several NPs can improve the photosynthetic system. The delivery of NMs is directed at either the plant roots or vegetative parts, whereas the primary focus is the leaves (Usman et al., 2020). The leaf lamina penetration approach could enhance NP infiltration in plant tissues (described for single-walled CNTs) and has proven to be applicable for gene delivery (Giraldo et al., 2014; Demirer et al., 2019b). Nanotechnology could help in developing faster manufacturing and industrial processes. The major tropical crop farmers, such as rubber, chocolate, coffee, and cotton farmers, will accomplish new goals that could lead to a new and enhanced nanoeconomy. GM crops will contribute to the new levels of sugar stopping, providing customers with many options. A programmed and centrally regulated industrial and agricultural sector can now be fulfilled via molecular sensors, automatic distribution systems, and low-cost technologies.

NPs have been used in various plants, including fruits, such as *Avena sativa* L. (Armendariz et al., 2004), blackberry (Nadagouda et al., 2014), *Citrus sinensis* L. (Sujitha and Kannan, 2013), olive (Khalil et al., 2012), and pear fruit (Ghodake et al., 2010). In particular, nanocalcium improves “Red Delicious” properties in apple fruit (Ranjbar et al., 2020). Recently, MNPs have been identified with antifungal properties that could be utilized in various fruit-bearing tree plants, including apples, pears, grapes, and citrus fruits, as well as other industrial crops. MNPs can also serve as biosensor particles to detect various biochemical disruptions in plants and humans (Thakur et al., 2020). The functional utilization of NPs is not limited to specific crops; moreover, NPs have a wider utilization and adoptability from medicinal and industrial crops to fruits and woody trees.

## Challenges in GE and NP-Mediated GE in Plants

The GE technique modifies plant cell genomes, involving the efficient delivery of modifier biomolecules as genetic cargo to targeted plant cells (Demirer and Landry, 2017; Nandy et al., 2020). However, the available biomolecule cargo delivery techniques are non-efficient, causing a lag in genetic transformation. Moreover, these methods have several limitations that hinder robust GE because of non-specific site integration, damage to plant tissues, non-significant gene expression after integration, tissue specificity, and species specificity (Altpeter et al., 2016). These techniques are available for the narrow host range and cause postmodification regeneration and fertility problems in transgenic plants. Methods, such as AMT and gene gun-based transformation, have certain limitations of use, making them non-versatile for general use. However, they are now well-established and have produced numerous successes. With the development of multiplex GE using the CRISPR/Cas9 technique (Ma et al., 2015), research on GE has been significantly progressed. However, certain complications remain, which limit the robust delivery of genetic cargos. One of the main obstacles here relates to how plant cells have an additional cell wall compared with animal cells, which provides them with rigidity, definite shape, and growth potential while acting as a physical barrier from environmental conditions (Cosgrove, 2005). The delivery of biomolecules to plant cells for GE remains a bottleneck owing to the physicochemical properties of the cell wall (Azencott et al., 2007). A plant cell wall mainly contains complex polysaccharides with a pore size varying from 3.5 to 5.2 nm, which provides rigidity (Carpita et al., 1979). Because of this narrow pore size and rigid structure, many genetic cargos cannot pass through it. Although AMT is widely used for genetic transformation, its efficiency depends largely on the host species and leads to undesired DNA integration in the host genome (Baltes et al., 2017). In view of the abovementioned issues, NPs have emerged as the best genetic cargo material because of their ease of use and success in several cases (Cunningham et al., 2018; Wang et al., 2019).

The unmatched potential of the NP-based delivery of biomolecules to plant cells (Deng et al., 2019) has revolutionized the GE delivery process (Deng et al., 2019; Landry and Mitter, 2019). In this method, the NP-bound GE nuclease is efficiently transferred to plant cells without causing damage to the target tissue. The use of NP-based methods instead of the conventional methods of genetic cargo delivery has emerged as a part of a cutting-edge technology that provides new insights and a robust GE. The NP-mediated transfer of biological molecules to plant cells has abrogated all issues previously hindering the success of GE, and it thus presents a promising technique for enhancing the efficiency, robustness, and versatility of GE (Cunningham et al., 2018). Due to their small size, NPs can transverse the cell wall and overcome barriers to delivering biomolecules to plant cells.

**FIGURE 4:**
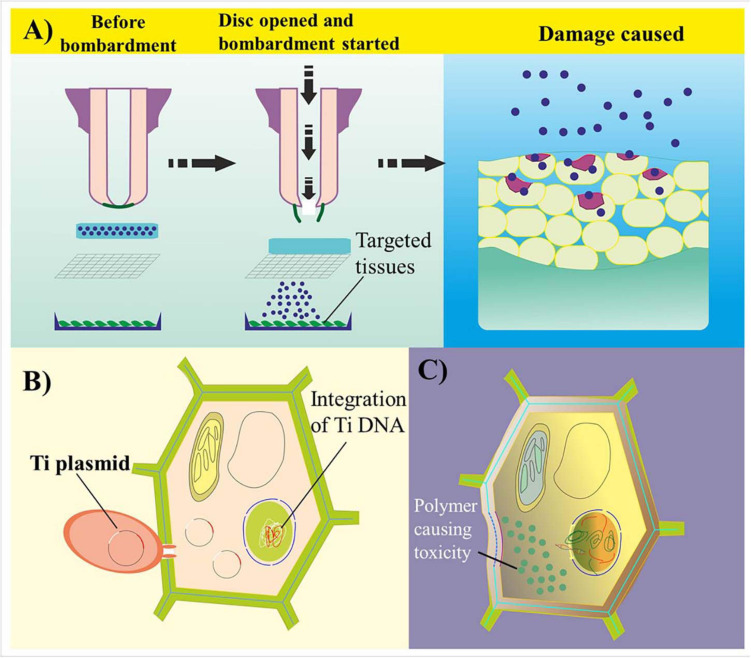
Common challenges in genome editing- and nanoparticle-mediated plant transformation. **(A)** Biolistic delivery of biomolecule-coated particles into targeted plant cell tissues. Because of the unavoidable high velocity of genetic cargo, the bombarded particles damage the cell wall through penetration and disrupt cell homeostasis. **(B)** Transformation of plant cells via *Agrobacterium*-mediated transformation. The T-DNA of *Agrobacterium* integrates within the host genome, causing a tumor or a change in the genetic information of the transformed cells. **(C)** Polymer-based transformation leads to cytotoxicity in plant cells because of the accumulation of high-density charged polymer-based genetic cargo. A reduction in charge leads to an impairment in the bioconjugated complex.

Despite its significant importance, certain challenges are hindering the effective use of NPs in GE. The first relates to nanophytotoxicity (Cox et al., 2016). Nanophytotoxicity is defined as the negative effect of NMs on plant growth, causing damage to either the plant or the environment because of the subsequent release of NMs up to a toxic level (Figure 4). Various studies have demonstrated that the uptake of NPs by plants results in some phytotoxicity due to the blockage in the plant vascular system, resulting in structural damage to the plant’s DNA and inducing oxidative stress (Pachapur et al., 2016; Du et al., 2017; Rastogi et al., 2017). The reproductive growth of plants is also negatively regulated by the toxicity of silver NPs (Dutta Gupta et al., 2020). Nevertheless, increases in leaf and root growth as well as improved chloroplast production have been observed after NP-based transformation (Cox et al., 2016; Zuverza-Mena et al., 2017). the translocation, deposition, and culture of nanotoxic-free plants in subsequent generations need to be addressed. Here, although the amount of engineered NPs required as genetic cargo is significantly less than the toxic level in terms of both the environment and the plant, their deposition and dispersal to other plant cells after application require further research.

Different NPs can behave very differently in specific plant cells, which require optimizing their application for different plant species and their dose and spatiotemporal tuning. In plant cells, NM deposition results in extra reactivity, dynamic transfer to other plant parts, and instability (Lv et al., 2019). Several NPs have high oxidative properties, which lead to a disturbance in normal cell metabolism and interfere with the genetic regulation of plant cells, resulting in the oxidative rupture of the transformed cells (Hossain et al., 2015; Du et al., 2017). Their optimization for successful use as genetic cargo for the successful application in plant cells is crucial. At the same time, there should be no or, at least, minimum interference from NPs in cellular processes. Cell structural stability and metabolic pathway disturbance is another challenge that needs to be researched to improve the use of NPs as genetic cargo (Hossain et al., 2015; Lowry et al., 2019). However, studies have reported an efficient delivery of biomolecules for gene silencing in plants with no toxicity and no physiological disturbance or metabolic hindrance after CNT-mediated gene silencing and transient expression in mature plants (Demirer et al., 2018). Given that plant species and their tissues have different cell structures, the broad-spectrum application of NP-mediated delivery remains a challenge.

Another challenge for NP-mediated GE’s efficacy relates to the efficient binding of biomolecules to NPs and the disintegration of the binding complex in plant cells (Saptarshi et al., 2013; Fleischer and Payne, 2014). Different biomolecules have a different binding affinity with other NPs based on their structure, charge, chemical composition, and surface area, making them ideal for a bioconjugation complex. The interaction between EMNs and plants largely depends on intrinsic properties, such as chemical composition, spatiotemporal occurrence, shape, size, hydrophilic or hydrophobic nature, and crystalline structure (Nel et al., 2009; Dasgupta et al., 2014). NPs can also be charged, and their surface can be designed to bind with diversely shaped biomolecules and hence can be an excellent platform for delivering biomolecules (Dasgupta et al., 2014; Hu et al., 2020). Moreover, NPs can be engineered to mediate cargo delivery to any subcellular parts that AMT cannot target, such as mitochondrial or chloroplast DNA. They can also be used without the species- and tissue-specific limitations of the previously available biomolecule delivery methods. However, their optimization for binding specific biomolecules requires further research to enhance their versatility as genetic cargo.

For the promising future of NP-mediated GE, scientists have attempted to understand how an NP-biomolecule bioconjugated complex will be delivered in a force-independent manner (Busch et al., 2019). Such nanocarriers have been studied for their specific delivery to plant organelles without damaging the transformed cells and having the least residual effect on the daughter cells with no toxic impact on the plant or the environment (Hu et al., 2020). Plant nanobiotechnology is an emerging field and requires input from all scientific fields, including biochemistry, molecular biology, biophysics, and structural chemistry. After optimizing the dose, the delivery method, and the NP type, it will be possible to establish a complete revolution in delivering genetic cargo based on nanocarriers.

## Speedy Crop Improvement Coupled With the Application of Nanobiotechnology

**FIGURE 5:**
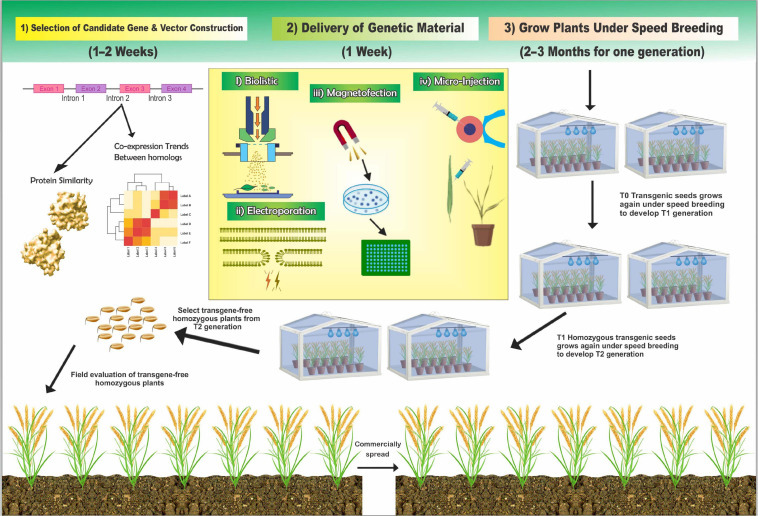
Major steps to efficiently improve the speedy crop process by combining the existing technologies.

A speedy crop can be produced using CRISPR under speed editing strategies by incorporating NMs to combine with speed breeding. One significant issue of the CRISPR gene-editing technologies for agricultural applications is that transgenic plants must be free of target genetic alteration to preserve the stability of the traits and secure regulatory clearance for commercial development (He and Zhao, 2020). Nanotechnology can help distribute genetic materials to plants to enable genetic engineering and stabilize genetic materials, including improving their double-stranded RNA efficacy for plant improvement (Hofmann et al., 2020). Furthermore, NMs used for grafting can be leveraged, and relevant biomolecules can be subsequently delivered for GE via plant cells owing to the difficulties in transporting exogenous biomolecules across cell walls (Wang et al., 2019). Demirer et al. (2019b) devised a tool for the species-independent, targeted, and passive delivery of genetic materials into plant cells without transgene integration for diverse plant biotechnology applications. Here, the authors demonstrated the efficient diffusion-based biomolecule delivery of CNTs into several mature plant species with a suite of pristine and chemically functionalized high aspect ratio NMs. Efficient DNA delivery and strong transient protein expression were accomplished in mature *Eruca sativa* (arugula-dicot) and *T. aestivum* (wheat monocot) leaves and protoplasts. In addition, Demirer et al. (2019b) demonstrates a second NP-based strategy in which low interfering RNA (siRNA) is delivered to mature *Nicotiana benthamiana* leaves to silence a gene with 95% efficiency. The developments in plant transformation include the delivery of DNA using polyethyleneimine-coated iron oxide MNPs as carriers and the application of a magnetic force to direct the MNP–DNA complexes into the pollen of cotton before pollination (Zhao et al., 2017). NPs can potentially deliver gene-editing cargos to any plant cells, including meristematic cells (Mohamed and Kumar, 2019; Sanzari et al., 2019; Wang L. et al., 2020). The delivery of GE reagents via NPs into meristematic cells can potentially generate chimerically edited plants. Transgene-free and edited plants can be regenerated from the edited tissue via tissue culture or by propagating cuttings. Elsewhere, a recent exciting report indicated that plasmid-coated carbon dots could be delivered into plant cells via foliar application (spraying on). The Cas9/gRNAs produced via this method successfully edited target genes (Doyle et al., 2019). The development of nanobiotechnology has presented new ideas for transgenic approaches using NPs as the gene carriers. However, it remains challenging to establish GM crops quickly and easily. This obstacle can be overcome by using a combination of existing technologies. A speedy crop improvement has been proposed as the best strategy for addressing these challenges. However, it is unclear how this speedy crop improvement process will work. In fact, there are four steps to perform a speedy crop breeding. First, we can select the best candidate(s) using speed editing strategies (CAFRI-Rice)^1^ based on protein sequence similarity and coexpression trends among homologous candidate genes with functional redundancy to enable more efficient multiple GE. This online tool remains limited to rice but will soon be updated for other crop species, with the process requiring a maximum of a single day (Ahmar et al., 2020a; Hong et al., 2020). Second, after the selection of candidate genes, the delivery of genetic materials (CRISPR binary vector) can be performed using a different type of nanotube or a different type of delivery method according to the lab facilities. This will take a maximum of 2 weeks (Zhao et al., 2017; Giraldo et al., 2019; Zhang et al., 2019a; Chandrasekaran et al., 2020; Wu et al., 2020). Third, after delivering the genetic/CRISPR vector, the transgenic plant will be grown under a speed breeding protocol to obtain the T_0_-generation seed. In the final step, T_0_ should be grown for T_1_ under speed breeding conditions to achieve T_2_ generation or segregation to develop transgene-free plants (Figure 5). The time required for this step varies depending on the plant species. However, four to six generations per year can be achieved, and the seed and plant density can be increased to efficiently scale-up the plant numbers using the single-seed descent method (Chiurugwi et al., 2018; Watson et al., 2018). The speed breeding protocol optimizes the rapid growth of oat, various *Brassica* species, chickpea, pea, grass pea, quinoa, and *Brachypodium distachyon* crops (Hickey et al., 2017; Ghosh et al., 2018; Jähne et al., 2020). This new strategy can be potentially extended to other plants, thereby offering a simple, fast, and inexpensive method for editing plant genomes.

## Future Directions

A revolution is needed in agriculture science to ensure that the agricultural sector is more effective, robust, and sustainable, and nanotechnology will play a critical role in designing smart crop systems. These smart crop systems will help address the food storage issue, which is a major global challenge. NPs can be successful in transmitting micronutrients or providing insect or pathogen protection mechanisms by increasing the amount of enzyme and non-enzymatic compounds as well as genetic supplies. Moreover, NMs can help increase crop quality and performance by reducing production costs and postharvest losses via nanograms, nanofertilizers, nanopesticides, nanosensors, nanobags, and nanochips. Furthermore, NMs will reduce the amount of sprayed agrochemicals and increase their efficacy through the intelligent supply of active ingredients and the reduction of nutrient losses during the fertilization process.

The GE field is highly complex because of significant obstacles to effective genetic transformation, including the issue of DNA transport through the plant cell walls and subsequently via the nucleus. In addition, the use of site-directed techniques for GE, such as CRISPR/Cas9, tends to be unreliable in terms of plant improvement.

The CRISPR/Cas9 technique entails various issues, including the off-target effects, high costs, low security, and device delivery and editing inefficiencies, that must be resolved in the current system. The development of new nanovehicles serving as molecular transporters could become a core catalyst for the genetic transformation of plants. It could play a key role in determining the delivery methods and enhancing the efficiency of transformation. The different types of NPs, such as hybrid NPs, graphene oxide NPs, peptide-based NPs, and nanogels, could facilitate the GE process of the CRISPR/Cas9 system. A potential natural CRISPR carrier exists for specific inorganic NPs, such as gold NPs, CNTs, MSNs, and dense silicon NPs, that could be used for relevant applications, such as those discussed above.

Despite the exceptional ability of NPs to introduce CRISPR/Cas9, there remain significant challenges that need to be resolved, including scale-up problems, poor encapsulation, bioprotection, continuous expression, and low transfection rates. However, the attendant distribution in cells and the possibility of editing the genome in the cells’ nucleus are key issues that can affect the success or efficiency of plant transformation. In particular, although the application of NMs within the CRISPR/Cas9 device distribution is superior to the previous delivery strategies in all fields of crop science, further testing is needed to ensure that the CRISPR method is delivered powerfully and reliably and is utilized in the short term. The CRISPR/Cas9 system in combination with NPs will provide a breakthrough in plant genetics in terms of testing other biological systems. Furthermore, previously developed technologies, such as speed breeding and speed editing strategies, could be developed to speed up the GE process that incorporates NPs and rapidly establish speedy crop improvements with the desired traits. Rapidly advancing technologies are undoubtedly providing plant scientists with new directions for overcoming the challenges of agricultural food supply faced throughout the world. This will also help develop new varieties, playing an important role in developing transgene-free plants using GE.

The widespread use of NPs has drawn public interest given that the food supply could be polluted by metal-based NPs. Here, zinc oxide NPs are among the most widely researched metal-based NPs in human and ecosystem health as well as plant nanotoxicology (Chen et al., 2015; Van Aken, 2015; Zhang et al., 2015; Tripathi et al., 2017). In earlier studies, the different impacts of metal-based NPs have been observed in plants, including those related to robustness, potential harm, or a non-influence (Millán-Chiu et al., 2017). However, most of these research has discussed easily observed parameters, such as the rate of germination and development. Plant responses to metal-based NPs are based not only on dosage but also on the plant species (Ghosh et al., 2019). Therefore, it will be in the best interest of all to focus on potential strategic studies for improving a regulated NP synthesis via a greener process and to gain a detailed understanding of a large number of unidentified NPs formed by the fungi and endophytes of the roots, which could play an important role in plant productivity. In this context, various organizations with the necessary skills and facilities that conform to the NP biosafety evaluations could be established. These organizations could operate in an interconnected manner to appropriately track the experimental findings for chemical and biological research institutes. Meanwhile, the global food protection and standard authorities should strictly comply with the Food and Agriculture Organization/World Health Organization standards and specific recommendations for monitoring or assessing NP-based systems. Furthermore, all nanobased foods should be tested to address any safety concerns before their commercial introduction, whereas corresponding data from many samples should be collected. Meanwhile, the scientific community as a whole should be encouraged to use multiple digital programs related to the possibilities and functioning of nanotechnology. Focusing on the essential facets of plant physiology can, of course, be expanded to include various identified applications. The delay in the advancement of plant nanotechnology could be resolved by promoting multidisciplinary approaches to the intelligent design and synthesis of NMs. However, the improvement of NPs or microparticles and the distribution methods for biolistic gene transfer in different plants are still required to enhance seed growth and improve plant and crop protection.

## Conclusion

This review critically examines the various NP-mediated transgenic delivery strategies and the existing method-congested field of plant biotechnology. Here, we propose that more exciting techniques could be incorporated in the processing of modifying crops, such as the CRISPR technique, alongside a combination of the several recently developed technologies. However, several major issues still need to be resolved. Most of these issues could be addressed by integrating different solutions for the effective delivery of different genomes, the design and fabrication of modern hybrid NMs, and the improvement of pollen magnetofection and CRISPR strategies. Overall, the food and farming sectors of the future should perhaps not be a concern because, while the nanotechnology applications may take some time to enter the field, the continued support and awareness of these issues will ensure that the field will continue to grow and develop.

## Author Contributions

SA and K-HJ conceived and designed the article. FM-P, TM, MSS, SF and MSC contributed to the manuscript revision. SA and K-HJ, FM-P, TM, MSS, SF and MSC wrote the manuscript and supervision FM-P and K-HJ. All authors contributed to the article and approved the submitted version.

## Conflict of Interest

The authors declare that the research was conducted in the absence of any commercial or financial relationships that could be construed as a potential conflict of interest.

## Footnotes

1 http://cafri-rice.khu.ac.kr/
